# Impact of Day/Night Cycle Temperature Regimes on Zika Virus Replication in Mosquito Cells and Implications for Transmission Potential

**DOI:** 10.3390/pathogens15080839

**Published:** 2026-08-12

**Authors:** Breanna R. Timani, Rachel N. Robertson, Katherine D. Shields, Mekala Sundaram, Melinda A. Brindley

**Affiliations:** 1 Department of Infectious Diseases, College of Veterinary Medicine, University of Georgia, Athens, GA 30602, USA; breanna.timani@uga.edu (B.R.T.); rachrob1245@gmail.com (R.N.R.); katherine.shields@uga.edu (K.D.S.); 2 Savannah River Ecology Laboratory, University of Georgia, Athens, GA 30602, USA; 3 Department of Infectious Diseases and Center for Precision One Health, University of Georgia, Athens, GA 30602, USA; 4 Department of Infectious Diseases, Department of Population Health, College of Veterinary Medicine, University of Georgia, Athens, GA 30602, USA

**Keywords:** Zika virus, mosquito-borne virus, temperature, disease transmission model

## Abstract

ZIKV is an emerging mosquito-borne pathogen with the unique capability to cause severe congenital abnormalities. Mosquito-borne viruses are reliant on an optimal temperature within the mosquito host to spread to humans. Few studies explore how day/night temperature variations impact virus replication within the mosquito. Most laboratory studies are conducted at constant temperatures, or with very minimal temperature oscillations. In consequence, ZIKV transmission models and risk maps utilize biological data derived from constant temperature studies. We sought to determine if the use of viral replication data obtained in more biologically relevant, fluctuating temperatures alters the geographic range for ZIKV transmission risk. We used a growing degree day (GDD)-based model to determine ZIKV transmission potential across the US. Replication curves were conducted at eight different average temperatures between 18°C and 32°C with daily fluctuations of either +/−2.5°C or +/−5°C from the baseline. We found that at lower temperatures (18–22°C), fluctuation was beneficial for virus replication, where it had less of an impact at more optimal temperatures (24–30°C). Our transmission potential map exhibited the highest proportion of infected mosquitoes in southeastern United States, with discrete pockets of elevated potential across interior regions, including the southern Great Plains and lower Mississippi Valley.

## 1. Introduction

Zika virus (ZIKV) emerged as a major global health concern during the 2015–2016 outbreak in the Americas, resulting in over 500,000 confirmed cases, with many more cases suspected [1,2]. The outbreak’s most devastating consequence was the association between maternal ZIKV infection and severe birth defects, namely microcephaly, in developing fetuses. Although the global burden of ZIKV has decreased, the effects of urbanization and climate change have unlocked the potential for ZIKV to reemerge as a public health threat in the future [3,4,5,6,7,8]. Understanding ZIKV transmission dynamics is therefore essential for anticipating and controlling future outbreaks.

ZIKV is transmitted by infected *Aedes aegypti* and *Aedes albopictus* mosquitoes. The transmission cycle begins when a mosquito vector takes a blood meal from an infected mammalian host. The virus replicates in the midgut and then spreads to the salivary glands over the course of 1–2 weeks, allowing the mosquito to become infectious and capable of transmitting the virus to the next host [9,10,11]. The viral replication process is critically dependent on environmental temperature [12,13]. Mosquitoes, being ectothermic organisms, cannot regulate their own body temperature and instead equilibrate with their surrounding environment. Throughout each day, a mosquito’s body temperature will vary with its environment. Temperature directly influences the rate and efficiency of viral replication within the mosquito’s cells [12,13]. This relationship between temperature and viral replication determines the extrinsic incubation period (EIP) —the time required for a mosquito to become infectious following ingestion of an infected blood meal [14,15]. In warmer conditions, the EIP shortens, meaning mosquitoes become infectious faster and can transmit the virus sooner [13,14,15,16]. Conversely, cooler temperatures extend the EIP, slowing viral spread and reducing transmission efficiency [13,14,15,16]. This temperature-dependent mechanism has profound implications for predicting where and when ZIKV outbreaks are likely to occur.

Previously we observed that ZIKV transmission in mosquitoes is optimal between 28 and 32°C, with a viable thermal range spanning 22.7°C to 34.7°C [13]. Below 22°C, virus replication is limited, while temperatures above 34°C result in rapid mosquito death [13]. Our previous study, like the majority of laboratory studies monitoring temperature effects, employed constant temperature conditions [12,17,18,19,20], which do not reflect natural environmental variability. We found ZIKV replication in mosquito cell cultures at various constant temperatures closely aligned with our data in the mosquito, with ZIKV genome replication being limited at temperatures below 20°C [12]. Notably, this inhibition could be overcome by first incubating the cell culture above 20°C for 12 h, allowing the virus to overcome RNA replication inhibition [12]. This observation suggested that viral replication dynamics might differ under conditions that a mosquito would experience in nature when subjected to day/night fluctuations compared to the constant temperature regimens typically used in laboratory studies.

Few studies have examined the impact of natural day/night temperature cycles on ZIKV replication and transmissibility [10,21]. This gap in understanding represents a significant limitation, as laboratory constant temperature conditions fail to capture the thermal dynamics that mosquitoes encounter in their natural habitats. Consequently, current ZIKV transmission models and risk maps primarily use two sources of biological data to determine how ZIKV behaves under varying temperatures: ZIKV laboratory data from experiments at constant temperatures or data from Dengue (DENV) transmission at constant temperatures [6,13,22,23]. In previous transmission models, empirical EIP data for ZIKV or DENV across multiple constant temperatures was used to determine the EIP in different regions based on the area’s average temperature [6,13,22,23]. As these EIP estimations are based on constant temperature data, and do not consider daily temperature variation, they may underestimate how the ZIKV EIP is impacted by temperature variation in nature. Similarly, other parameters such as mosquito development, hatching rate, biting rate, and death rate could also differ under daily temperature variation. Given these methodological constraints, existing risk maps may substantially underestimate the true transmission potential of ZIKV. Specifically, regions with subtropical climates and regular temperature variation may be subject to greater ZIKV replication than expected within mosquitoes in that climate. Regions where ZIKV is endemic range from an annual average of 24–26°C, and areas susceptible to seasonal ZIKV outbreaks may have an annual average as low as 20 C, but with yearly fluctuations of 10°C [22].

To address this gap, we examined ZIKV replication in C6/36 *Aedes albopictus* cells at constant temperatures compared to day/night oscillating conditions around multiple average temperatures within the range of 18–32°C. After determining the effects of daily temperature changes on ZIKV replication in mosquito cells, we integrated this data into a growing degree day (GDD)-based transmission potential model. Growing degree days track the accumulation of temperatures experienced by mosquitoes over time, reflecting the thermal conditions required for vector development and viral replication [24,25,26]. GDD models have been successfully applied to characterize within-vector dynamics of West Nile virus [25,26,27], but have not been used to assess within-host ZIKV accumulation dynamics.

Integration of empirical viral amplification data into vector-borne disease models is well-established in the SIR/SEIR literature [6,13,22,23]; here, we extend this approach by embedding viral titer accumulation, expressed as GDD, directly into an agent-based model of *Aedes aegypti* population dynamics. This allows us to simultaneously evaluate both the thermal suitability for mosquito population persistence and the likelihood of ZIKV reaching replication thresholds sufficient for transmission, across 2578 weather stations spanning the United States on 1 August 2025. Rather than treating viral replication as a fixed parameter, this framework captures how day-to-day temperature variation shapes both vector population dynamics and within-vector virus growth, providing a more realistic assessment of spatial transmission risk than mean-temperature approaches alone. The objective was to determine the geographical ranges where there is a higher risk for ZIKV transmission with a model using GDD-based, fluctuating temperature-derived data that more accurately reflect natural conditions. We then compared our findings to previous ZIKV risk models that utilized constant temperature laboratory data.

## 2. Materials and Methods

### 2.1. Cells

C6/36 mosquito cells (ATCC CRL-1660) derived from *A. albopictus* larvae were maintained at 28°C in Leibovitz’s L-15 medium supplemented with 10% fetal bovine serum (FBS). For replication curves utilizing colder temperatures, 18°C, 20°C, and 22°C +/− 2.5°C or +/−5°C, a cold-adapted C6/36 cell line previously generated in the lab was used [12]. Vero (African green monkey kidney) cells used for virus titration were maintained at 37°C in Dulbecco’s modified Eagle medium (DMEM) with 5% FBS and 5% CO_2_. All cell cultures tested negative for Mycoplasma.

### 2.2. Viruses

ZIKV MEX1-44 was isolated from a field-caught *A. aegypti* mosquito in Chiapas, Mexico, in 2016 and was obtained from the University of Texas Medical Branch [28]. Viral stocks were generated and titrated in Vero cells. Stock titers were determined by endpoint dilutions on Vero cells. The 50% tissue culture infectious dose (TCID_50_) was determined using the Spearman-Karber method [29].

### 2.3. ZIKV Replication Curves

Viral replication curves were produced in 12-well plates in C6/36 cells (4 × 10^5^ cells/mL). C6/36 cells were infected with a multiplicity of infection (MOI) of 0.1 for 2 h under constant conditions. Viral inoculum was removed and fresh media were replaced; then, supernatants were collected for the 0 h time point samples, and plates were moved to their respective temperature regimen. Supernatants were collected at each time point indicated and frozen at −80°C, and fresh media were replaced. Upon completion of the time course, supernatants were thawed, and viral titers were determined by endpoint dilutions on Vero cells, followed by TCID_50_ calculation.

### 2.4. Incubator Conditions to Mimic Day/Night Temperature Oscillations

ZIKV replication curve experiments were conducted in incubators (Percival Scientific, model I-30VL, Perry, IA, USA, 50220) at 90 ± 10% relative humidity with temperatures set to mimic the day/night cycle experienced by mosquitoes in nature. The temperature program used was developed by Paaijmans et al. [30]. Two incubators were used at once; both were set at the same constant temperature baseline per replication curve experiment, with one incubator having a diurnal temperature range (DTR) of 5°C (±2.5°C), and the other having a DTR of 10°C (±5°C) (program setup in Supplemental Materials). Constant temperature replication curves were conducted at the same time as fluctuating temperature experiments, using incubators set at the constant baseline temperature. Exact temperatures in each incubator were monitored using temperature loggers (OM-62; Omega, Eustache, QC, Canada, J7R 2H7) (Omega Logger Interface Program Version 1.41.0) at 30 min intervals and ensured the incubators were at the expected temperature.

### 2.5. Statistical Analyses

We analyzed laboratory data describing ZIKV amplification across temperature treatments in R (4.3.1) to quantify the relationship between viral titer and accumulated thermal exposure. We calculated growing degree days (GDDs) using the Type-C method (implemented in ‘pollen’ package) across candidate lower and upper temperature thresholds [31]. For each threshold combination, we evaluated the monotonic association between GDD and viral titer using Spearman rank correlation and linear regression (R^2^). We selected the threshold combination that maximized both correlation strength and model fit to parameterize GDD calculations in the agent-based model (Supplementary Materials Tables S1 and S2 [31,32,33,34,35,36]).

We implemented a stochastic agent-based model (ABM) in R to simulate mosquito population dynamics and ZIKV transmission potential under variable temperature-dependent processes (full methods in Supplemental Materials). We tracked individual mosquitoes by age, infection status, sex, accumulated viral titer, and reproductive output. We initialized the population with N = 1000 individuals, assigning ages from a uniform distribution (0–18 days), an equal sex ratio, and an initial infection proportion I_0_ = 0.05. We ran simulations for 50 daily time steps and capped population size at 1000 individuals to reflect carrying capacity constraints.

We derived temperature-dependent mortality from a Weibull parametric survival model (‘survival’ package [33]), generating daily age-specific mortality probabilities (ages 1–18 days). We implemented mortality as a Bernoulli process at each time step and assigned a mortality probability of 1 to individuals older than 19 days. We modeled reproductive output using a Weibull 1995 thermal performance curve (‘rTPC’ package [35]) fitted to empirical egg production data [34], with female offspring production following a temperature-dependent Poisson process and 10% of offspring recruited per time step. Notably, the mortality and reproductive output data are based on experiments using *A. aegypti* rather than *A. albopictus*, though our viral replication curves were conducted in C6/36 cells. *A. aegypti* is known to tolerate a wider and higher range of temperatures and maintains efficient pathogen replication at hotter tropical extremes, whereas *A. albopictus* is better adapted to cooler baseline conditions and experiences decreases in competence in hotter conditions [37,38,39]. While this difference in study species may influence the magnitude of predicted temperature effects, it is unlikely to alter the broader trends observed.

We modeled transmission stochastically by randomly infecting 5% of susceptible adults at each time step. Within-host viral replication is represented as cumulative GDDs calculated using station-specific daily minimum and maximum temperatures and thermal thresholds of 19°C and 31°C. We reverted individuals that failed to accumulate 100 GDDs to an uninfected status, representing the minimum thermal exposure required to complete viral growth. We defined the primary model output as the proportion of infected individuals at time step 50.

We obtained daily minimum and maximum temperatures for 1 August 2025 from 2578 US weather stations using the GSOD database (‘GSODR’ package [36]). We ran simulations independently per station and parallelized using a SLURM job array on the GACRC Sapelo2 HPC cluster. We mapped proportion of infected mosquitoes at time step 50 to characterize spatial variation in temperature-driven ZIKV transmission potential.

## 3. Results

### 3.1. Daily Temperature Variation Benefits ZIKV Replication at Lower Temperatures

**Figure 1:**
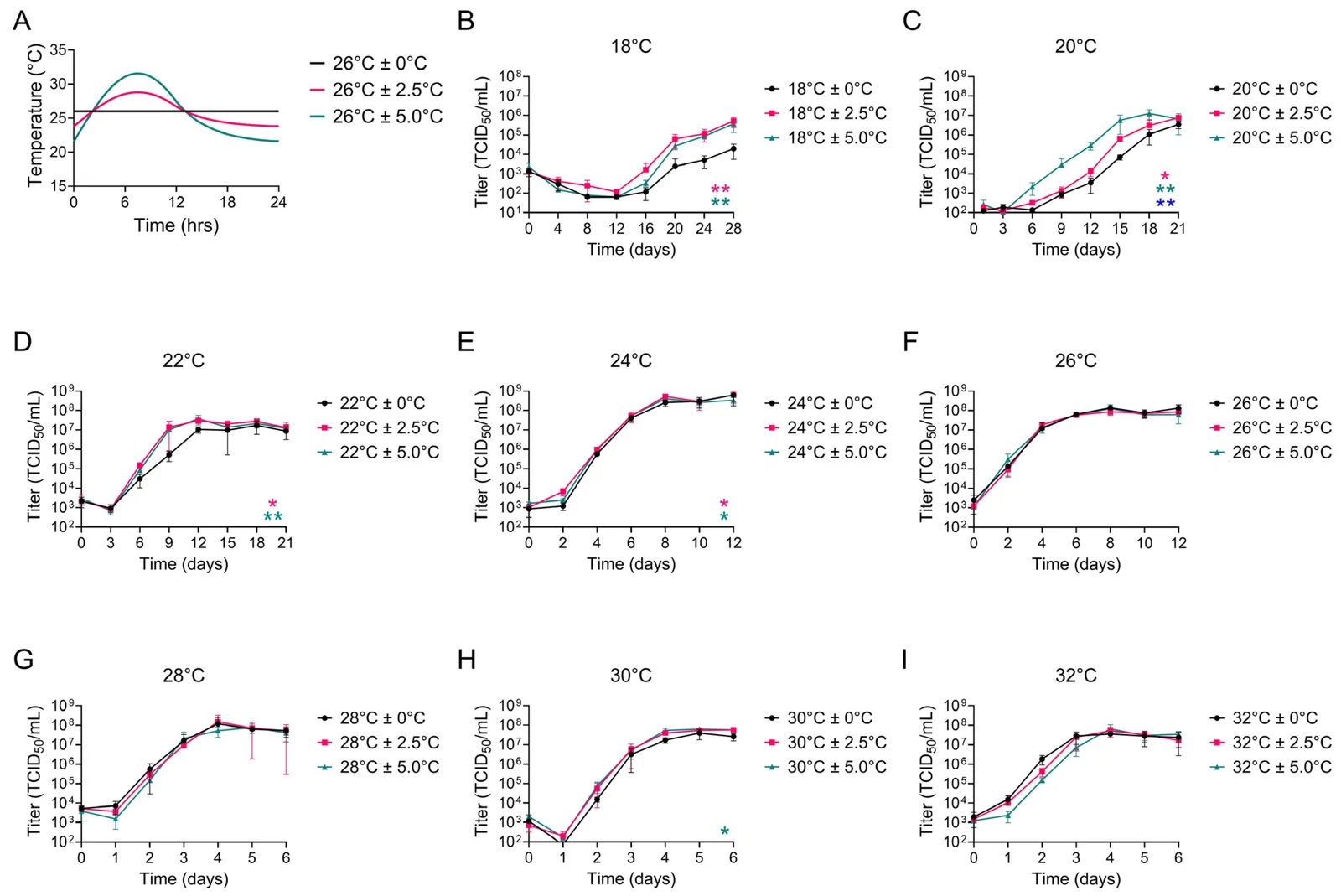
Viral replication curves in C6/36 cells. Replication curves were conducted under constant conditions, a DTR of 5 (±2.5°C), or a DTR of 10 (±5°C) from the average temperature according to a day/night cycle program. (**A**) Graph displays a representative temperature variation across a 24 h period. (**B**–**I**) Replication curves conducted at constant temperatures (black), ±2.5°C fluctuating conditions (pink), and ±5°C fluctuating conditions (teal) are displayed. Viral titers were determined by endpoint dilutions on Vero cells, followed by TCID_50_ calculation. Average titer and standard deviation are shown from three independent experiments. We used log transformed viral titer data to conduct non-linear fitting for logistic growth. Area under the curve (AUC) was used to compare replication curves. Student T-test comparing constant and ±2.5°C shown in pink, constant and ±5°C shown in teal, and ±2.5°C and ±5°C in blue. * *p*-value < 0.05; ** *p*-value < 0.01.

To determine whether temperature variation affects ZIKV replication dynamics differently than constant temperatures, we generated viral replication curves in C6/36 cells exposed to constant temperatures or two fluctuating temperature regimens (±2.5°C and ±5°C oscillations) at average temperatures ranging from 18 to 32°C (Figure 1A). At warmer temperatures (24–30°C), ZIKV viral titers peaked rapidly regardless of whether cells were maintained at constant or fluctuating environments (Figure 1E–H). At 32°C, we observed a modest reduction in virus replication under fluctuating conditions compared to constant (Figure 1I). In contrast, at cooler temperatures (18°C, 20°C, 22°C), temperature fluctuation substantially enhanced viral replication compared to constant temperature conditions (Figure 1B–D). Specifically, at 18°C, ZIKV replication was most efficient under the DTR of 5°C (±2.5°C) (Figure 1B). At 20°C, the DTR of 10 (±5°C) produced the highest viral titers (Figure 1C). At 22°C, both day and night cycle regimens (±2.5°C and ±5°C) enhanced replication equally compared to constant temperature (Figure 1D). At 24°C, AUC suggests that both day/night temperature regimes were significantly different than the constant condition; however, there were only differences very early in the curves (Figure 1E). Notably, across all three cooler temperature conditions, viral titers peaked substantially earlier under fluctuating temperatures than under the corresponding constant temperature condition (Figure 1B–D).

**Figure 2:**
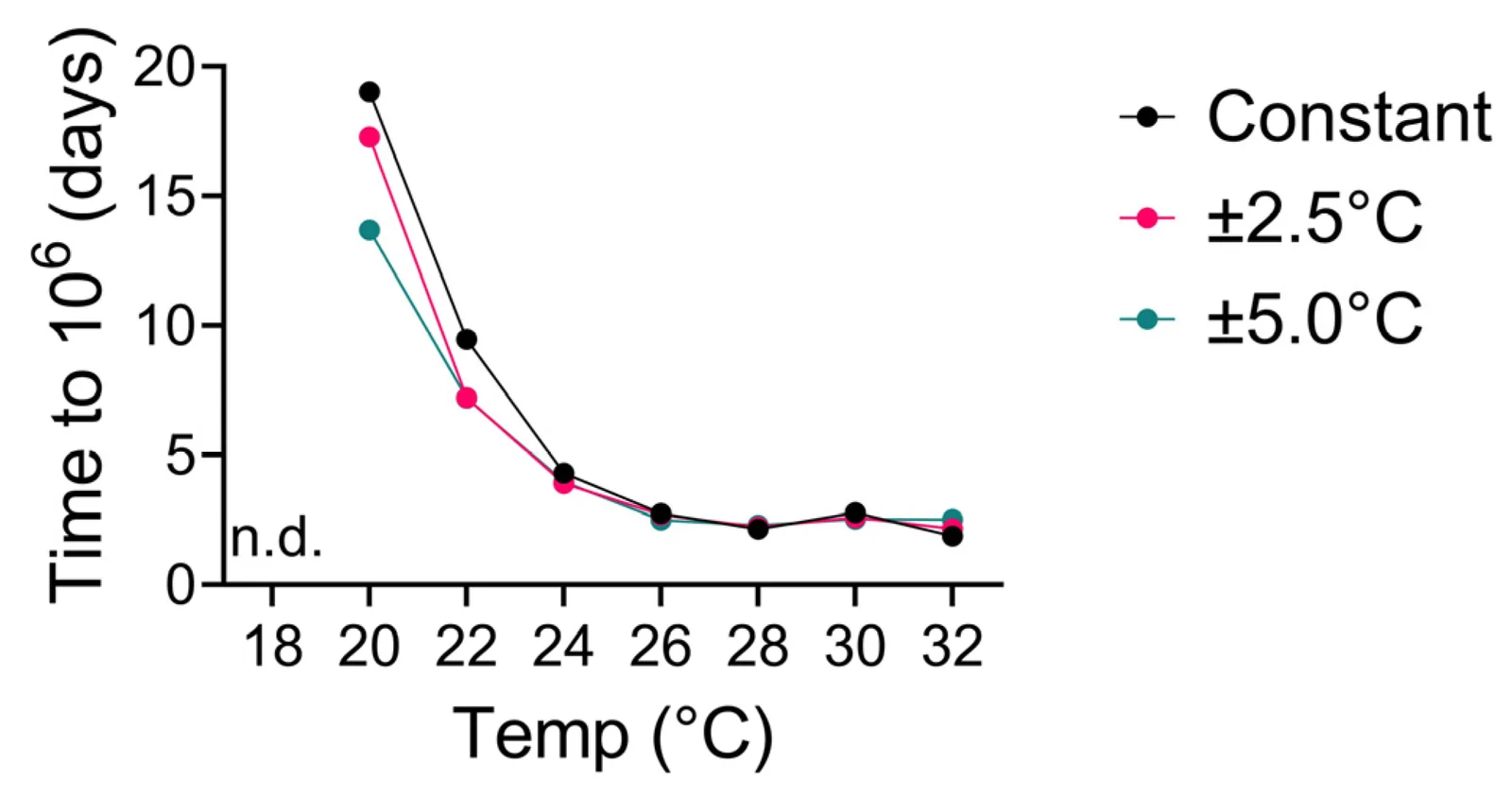
Time in days for viral titers to reach 10^6^ TCID50 units/mL. For each set of conditions (constant, ±2.5°C and ±5°C), we compare the days in which the viral titers reach 10^6^ TCID_50_ units/mL. We used log transformed viral titer data to conduct non-linear fitting for logistic growth. The following equation was used to determine the time when titers reached 10^6^ for each temperature group (solving for x): Y = YM × Y0/((YM − Y0) × exp(−k × x) + Y0). Statical analysis is presented in Supplemental Table S3.

Once we established differences in replication kinetics of ZIKV at constant versus fluctuating conditions, we wanted to succinctly display the overall trend of viral replication across temperature groups. We observed that, as temperatures increased, it took less time for ZIKV to reach peak titers (Figure 2 and Supplemental Table S3). At cooler temperatures (20°C and 22°C), we see that fluctuations decrease the amount of time needed for the virus to reach 1 million TCID_50_ units/mL (Figure 2). We were unable to conduct non-linear logistic regression using our 18°C data, as ZIKV replicated slower and never reached a peak under these conditions.

### 3.2. ZIKV Transmission Potential in the United States Based on a Growing Degree Day Model

**Figure 3:**
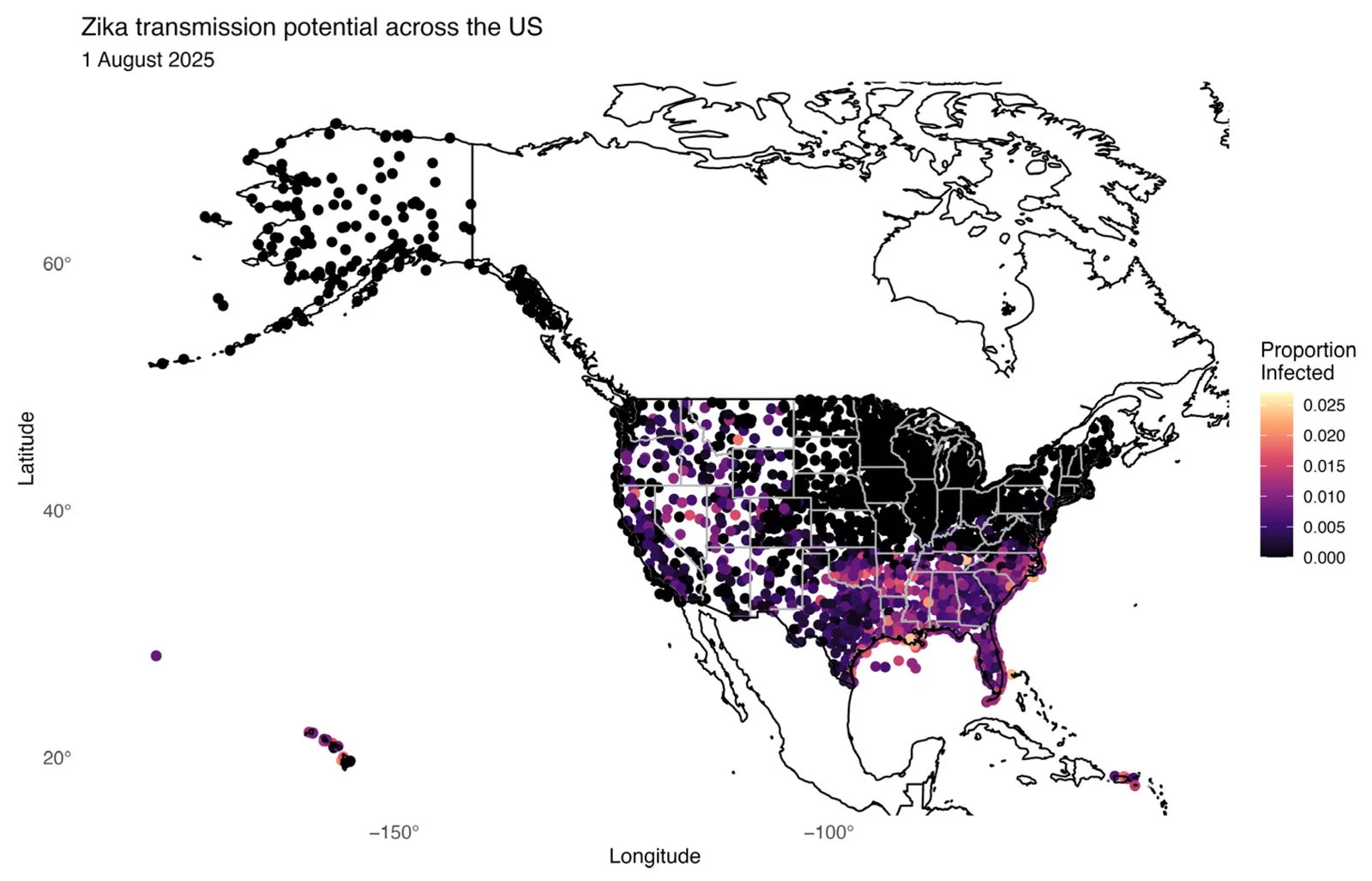
Spatial variation in the proportion of infected mosquitoes estimated from a stochastic agent-based model parameterized with observed daily temperature conditions at 2578 weather stations across the continental United States on 1 August 2025. Infection proportion reflects temperature-driven mosquito population dynamics and Zika virus replication potential based on growing degree day accumulation.

We next aimed to determine what ZIKV transmission potential in the United States would look like utilizing our ZIKV replication curve titers using the day/night oscillations (Figure 1). Viral titers were used to calculate the candidate lower and upper temperature thresholds for viral growth within the simulation. We selected the threshold combination that maximized both correlation strength and model fit to parameterize GDD calculations in the agent-based model. We empirically optimized GDD thermal thresholds and identified a lower threshold of 19°C and an upper threshold of 31°C, which produced the strongest association between GDD accumulation and observed viral growth (Spearman’s ρ = 0.843, *n* = 540, *p* < 2.2 × 10^−16^) (Supplementary Materials Tables S1 and S2). Using these GDD thresholds, we found that temperature-driven gradients strongly shaped spatial variation in the proportion of infected mosquitoes across the continental United States under 1 August 2025 conditions. The southeastern United States, including Florida, the Gulf Coast, and the South Atlantic region, exhibited the highest infection proportions (Figure 3). We also identified discrete pockets of elevated potential across interior regions, including the southern Great Plains and lower Mississippi Valley, where temperatures supported GDD accumulation above the viral growth threshold. In contrast, northern states, higher elevation areas, and Alaska exhibited negligible infection potential (Figure 3).

## 4. Discussion

In the viral replication curves (Figure 1), we see that replication is not impacted by day and night cycle variation at temperatures close to the optimal range for replication (26–30°C). ZIKV replicates faster and reaches high titers more quickly at higher temperatures (28–32°C). At lower temperatures (18–22°C), daily fluctuations benefit viral replication compared to constant temperatures. We believe that the variation in colder temperatures enhances replication because the time spent at higher temperatures during the day is sufficient to sustain viral replication, even when the cell culture also experiences periods of colder conditions. At 18°C specifically, we suspect that the ±2.5°C variation benefited ZIKV more than the ±5°C variation because the several hours spent at lower temperatures of 13–14°C were too restrictive of replication to be overcome by time spent at higher temperatures.

A few studies have demonstrated that fluctuations at cooler temperatures increase flavivirus transmission from mosquitoes [40,41]. In mosquitoes infected with DENV, a large diurnal temperature range (DTR) at a low temperature (20°C) increased the proportion of infected mosquitoes compared to constant 20°C [40]. When compared to a constant low temperature of 23°C, adult mosquitoes carried a higher ZIKV RNA load at a DTR of 23–31°C [41]. Some disease transmission models observe this trend as well [22,42,43]. It is estimated that ZIKV transmission cannot occur at constant temperatures less than 23°C, but that there could be epidemics in areas with an average temperature of 20°C if there is seasonal variation of 10°C [22]. For DENV, a thermodynamic model predicted that at mean temperatures less than 18°C, transmission increases as DTR increases [42].

A limitation of previous studies that explore the effects of temperature fluctuation on flavivirus replication and dissemination is that temperature oscillation only occurred around one baseline temperature or within a narrow temperature range [10,14]. Hugo et al. exposed ZIKV-infected mosquitoes to fluctuating temperatures that mimic the day and night cycle, though only around one average temperature of 28°C [10]. They observed that varying temperatures did not impact overall viral titers in the mosquito, but impacted viral dissemination to the wings and legs [10]. We similarly observed that temperature variations around 28°C do not impact viral titer, as the most significant effects can be seen at lower average temperatures of 18–22°C (Figure 1).

A limitation in our replication curves is that the fluctuating temperature program does not fully capture dynamic temperature changes in a natural day–night cycle across geographic regions, as temperatures in our incubators rose and fell very consistently according to the program (Figure 1A). For example, if the daily peak is higher for a longer period, it may increase mosquito mortality; if the daily low is too cold for too long, it may stall viral replication and midgut escape.

Our risk model represents a simple proportion of infected mosquitoes across the US given the temperatures on 1 August 2025. As the model does not factor in the range of *Aedes* mosquito vectors, or any social determinants of transmission risk, the map solely indicates where temperatures would be sufficient for a higher proportion of mosquitoes to be infected. Socioeconomic and ecological factors are often included in the development of mosquito-borne disease transmission models [13,22,23]. Most models also represent disease risk as the number of months where ZIKV transmission is possible, or general risk of transmission, across a geographic range [6,13,23,44]. Regardless of these differences in the representation of transmission potential, we see pockets of elevated potential in northern regions such as northern California (0.020) and southern Montana (0.020), as well as Western states like Nevada and Utah (0.010–0.020) (Figure 3). Other risk maps predict a low general risk of transmission in these regions [6,13,22,23,44]. This suggests that, although there are additional factors that make these regions less susceptible to ZIKV transmission, they may have suitable temperatures for ZIKV to circulate in mosquitoes during warmer months. Overall, our transmission potential map displays significant overlap with other risk maps of regions where ZIKV transmission would be possible. Florida, the Gulf Coast, and the South Atlantic region exhibited the highest infection proportions, which aligns with previous findings [6,13,22,23,44].

It is important to note that temperature alone does not determine ZIKV transmission dynamics. Socioeconomic factors, urbanization, infrastructure, and country-specific mitigation strategies play significant roles in estimating ZIKV transmission across regions [45,46,47,48,49]. In the United States, *A. aegypti* has a geographic range that extends from southeastern Texas and southern Arizona to southern California [50,51]. *A. albopictus* thrives in cooler environments, and its distribution reaches across the Southeast, Midwest, and into the Northeast [50,51]. Our model displays a notable proportion of infected mosquitoes across several Northeastern and western states, including Oregon, Montana, Nevada, and Utah, which are currently outside the known range of ZIKV mosquito vectors.

In this study, we determined how ZIKV replication is impacted by day/night cycle temperature variations that more accurately reflect natural conditions compared to the typical constant conditions utilized in laboratory settings. Although a few studies have observed how temperature variation impacts flavivirus replication, none have done so as extensively as demonstrated in this study, as we employed an extensive range of temperatures with day/night cycle oscillations of both 2.5°C and 5°C. We found, in line with previous studies, that fluctuations around cooler temperatures promote ZIKV replication. Considering these findings, we hypothesized that ZIKV risk maps generated based on constant temperature mosquito studies may underestimate the risk of ZIKV transmission. We produced a disease transmission potential model based on growing degree days as a determination of EIP to predict which regions across the US had suitable conditions for ZIKV replication within mosquito hosts. Our transmission potential map shows substantial agreement with other risk maps, with the southeastern US consistently displaying the greatest risk. Interestingly, we also observe small areas of elevated potential in a few northeastern and western states. The GDD-based model has not been previously used to predict ZIKV transmission dynamics, though it has been used for West Nile virus [25,27]. While the use of a GDD framework is novel for ZIKV, the model also differs from previous SIR/SEIR-based approaches in several key assumptions and parameterizations. Additionally, because our risk map represents only the estimated proportion of infected mosquitoes across the US as a function of temperature, it is not directly comparable to models that simulate human transmission dynamics or estimate overall disease risk. Further studies are needed to reveal whether current risk assessment models, which rely on constant temperature-derived EIP assumptions, adequately capture ZIKV transmission potential across diverse climates.

Since ZIKV is still endemic in many parts of the world, the virus could reemerge as a public health concern. Climate change is also expected to alter global temperature ranges and mosquito habitats, which may lead to an expansion of transmissible ranges [4,52]. Further biological studies are needed to determine how natural temperature environments impact ZIKV replication within mosquito hosts, and how a new temperature landscape will affect disease transmission in the future.

## Data Availability

The original contributions presented in the study are included in the article: https://doi.org/10.6084/m9.figshare.32814092. Further inquiries can be directed to the corresponding authors.

## Supplementary Materials

The following supporting information can be downloaded at: https://www.mdpi.com/article/10.3390/pathogens15080839/s1.
