# Supplementary material for: Impact of Day/Night Cycle Temperature Regimes on Zika Virus Replication in Mosquito Cells and Implications for Transmission Potential
Source: Pathogens. 2026 Aug 12;15(8):839. doi: 10.3390/pathogens15080839 (PMC13516181; doi:10.3390/pathogens15080839)
Supplement: Supplementary file 1 [file pathogens-15-00839-s001.zip › Supplementary methods Pathogens.pdf]

Supplementary Materials

Supplemental Methods

Statistical analysis of viral growth and thermal threshold estimation

Laboratory data describing Zika virus growth across multiple temperature treatments were analyzed in R to quantify the relationship between viral titre and accumulated thermal exposure. Viral titre measurements from replicate experiments were combined and related to cumulative growing degree days (GDD) [31].

GDD values were calculated using a single-sine method implemented in the gdd function from the *pollen* R package [31,32], across a range of candidate lower (base) and upper temperature thresholds. For each combination of thermal thresholds, cumulative GDD was calculated for each observation and matched to corresponding viral titre measurements.

To identify the most biologically relevant thermal bounds for viral replication, linear regression models were fitted relating viral titre (and log-transformed viral titre) to cumulative GDD for each candidate threshold combination. Model performance was evaluated using the coefficient of determination (R^2), and the strength of monotonic association between viral titre and GDD was assessed using Spearman rank correlation.

The optimal lower and upper temperature thresholds were selected as those maximizing both model fit (R^2) and correlation strength. These empirically derived thresholds were subsequently used to parameterize GDD calculations within an agent-based model, linking temperature exposure to cumulative viral titre. All statistical analyses were conducted in R 4.3.1, and full scripts are provided on figshare (https://doi.org/10.6084/m9.figshare.328140921).

Agent-Based Model framework

We implemented a stochastic agent-based model (ABM) in R to simulate mosquito population dynamics and Zika virus transmission potential under temperature-dependent processes. Individual mosquitoes were explicitly represented and tracked by age, accumulated viral titre (growing degree days), infection status, sex, and reproductive output. The population was initialized with N = 1000 individuals, with ages drawn from a uniform distribution between 0 and 18 days and sex assigned with equal probability. A proportion $I_0 = 0.001$ of individuals were randomly initialized as infected. Simulations were conducted in discrete daily time steps over 100 iterations. To reflect biologically realistic carrying capacity constraints, total population size was capped at 10,000 individuals, with new births only added when the population was below this threshold.

Mortality rates were derived from a temperature-dependent survival model fitted to empirical data using a Weibull distribution via parametric survival regression (survreg, R survival package) [33]. Daily mortality probabilities were predicted for ages 1–18 days at the mean daily temperature for temperature profiles, calculated as the average of daily minimum and maximum temperatures. At each time step, mortality was implemented as a Bernoulli process using age-

specific probabilities. Individuals aged 0 days were assigned zero mortality probability, while individuals older than 19 days were assigned a mortality probability of 1, ensuring removal beyond the maximum lifespan threshold.

Reproductive output was modeled using a temperature-dependent nonlinear function fitted to empirical egg production data [34] using the Weibull 1995 thermal performance curve (rTPC package, nls_multstart) [35]. The predicted egg production rate at the mean station temperature was used as the rate parameter λ of a Poisson distribution. At each time step, reproductive output was assigned to all female individuals via a Poisson process. Ten percent of total offspring across the population were added as new individuals, subject to the population carrying capacity cap of 1,000. New individuals were initialized with age 0, viral titre 0, uninfected status, and randomly assigned sex.

Transmission was modeled as a stochastic process acting on susceptible adult individuals (age ≥ 1 day). At each time step, 5% of uninfected adult individuals were randomly selected and assigned infected status. Following infection, within-host viral replication was represented as a cumulative thermal exposure process using growing degree days (GDD), calculated from daily minimum and maximum temperatures using lower and upper thermal thresholds of 19°C and 31°C respectively (pollen package) [32]. GDD accumulation was precomputed as a lookup table for ages 1–30 days at each station's temperature regime. Individuals with accumulated GDD at their respective age below a threshold of 100 degree-days were assumed not to sustain infection and were reverted to uninfected status, representing the minimum thermal exposure required to complete the extrinsic incubation period (EIP).

At each time step, individuals were updated sequentially through mortality, reproduction, infection and viral replication, and aging. Age was incremented by one day per time step. If the population went extinct during a simulation, all outputs were recorded as zero. Population-level outputs recorded at each time step included total population size, number of infected individuals, total deaths, and total births. The primary model output was the proportion of infected individuals at time step 50, calculated as the ratio of infected to total individuals. Stations where the population went extinct before time step 50 were assigned an infection proportion of zero.

The model was implemented in R using the data.table package for efficient individual-level state tracking. Temperature-dependent mortality and reproduction functions were parameterized using survival analysis and nonlinear least squares regression respectively. Random number generation was initialized with a fixed seed (set.seed(7)) to ensure reproducibility.

Temperature variation data were obtained from the Global Surface Summary of the Day (GSOD) database using the GSODR package in R [36]. Daily minimum and maximum temperatures for 1 August 2025 were extracted for weather stations across the United States. Stations with complete temperature observations were retained, yielding 2,578 stations. Station-specific temperature regimes were used to parameterize the agent-based model, allowing local thermal conditions to drive mosquito demography and viral replication. Simulations were run independently for each station, and the proportion of infected mosquitoes at time step 50 was used as a proxy for local Zika transmission potential. To enable efficient computation across all stations, simulations were parallelized using a SLURM job array on the Georgia Advanced Computing Resource Center

(GACRC) Sapelo2 high-performance computing cluster, with stations divided into 26 chunks of approximately 100 stations each. Outputs were mapped geographically to characterize spatial variation in temperature-driven Zika transmission potential across the continental United States.

Supplemental Results

Table S1. Spearman's rho quantifying the relationship between viral growth and cumulative growing degree days (GDD) across candidate lower (base) (Low T) and upper temperature (Max T) thresholds, calculated using the Type C growing degree day method. Comparable optimal thresholds were obtained using R^2-based model evaluation. Bold values indicate the highest spearman's rho.

Max T	27	28	29	30	31	32	33	34	35	36	37	38
Low T												
16	0.724	0.740	0.752	0.759	0.765	0.768	0.772	0.773	0.775	0.775	0.775	0.775
17	0.770	0.781	0.791	0.797	0.803	0.806	0.807	0.808	0.809	0.809	0.809	0.809
18	0.798	0.809	0.820	0.823	0.826	0.829	0.830	0.830	0.831	0.831	0.831	0.831
19	0.817	0.827	0.835	0.837	0.839	0.840	0.842	0.842	**0.842**	0.842	0.842	0.842
20	0.814	0.825	0.829	0.831	0.833	0.834	0.834	0.834	0.834	0.834	0.833	0.833
21	0.824	0.832	0.834	0.834	0.834	0.833	0.832	0.831	0.830	0.829	0.829	0.829
22	0.792	0.795	0.795	0.793	0.791	0.789	0.787	0.786	0.785	0.784	0.783	0.783
23	0.792	0.791	0.789	0.784	0.781	0.778	0.775	0.774	0.772	0.771	0.771	0.771
24	0.726	0.724	0.719	0.714	0.710	0.706	0.705	0.703	0.701	0.700	0.699	0.699
25	0.683	0.681	0.677	0.671	0.667	0.664	0.660	0.659	0.657	0.656	0.655	0.655
26	0.604	0.604	0.601	0.595	0.591	0.587	0.584	0.582	0.580	0.579	0.578	0.578
27	-	0.530	0.528	0.520	0.516	0.512	0.508	0.505	0.503	0.502	0.501	0.501

Table S2. R^2 values describing the proportion of variation in viral growth explained by cumulative growing degree days (GDD) across candidate lower (base) (Low T) and upper temperature (Max T) thresholds, calculated using the Type C GDD method. Comparable optimal thresholds were identified using Spearman's rho. Bold values indicate the maximum R^2.

Max T	27	28	29	30	31	32	33	34	35	36	37	38
Low T												
16	0.51	0.54	0.56	0.57	0.58	0.59	0.6	0.6	0.6	0.6	0.6	0.6
17	0.57	0.6	0.62	0.63	0.64	0.65	0.65	0.66	0.66	0.66	0.66	0.66
18	0.61	0.64	0.66	0.67	0.68	0.69	0.69	0.69	0.69	0.69	0.69	0.69
19	0.63	0.66	0.68	0.69	0.69	0.7	0.7	**0.7**	0.7	0.7	0.7	0.7

20	0.64	0.66	0.68	0.68	0.68	0.68	0.68	0.68	0.68	0.68	0.68	0.68
21	0.63	0.65	0.66	0.66	0.66	0.66	0.66	0.65	0.65	0.65	0.65	0.65
22	0.58	0.6	0.61	0.6	0.6	0.59	0.59	0.59	0.58	0.58	0.58	0.58
23	0.56	0.58	0.57	0.56	0.55	0.54	0.54	0.54	0.53	0.53	0.53	0.53
24	0.48	0.5	0.5	0.48	0.47	0.46	0.46	0.45	0.45	0.44	0.44	0.44
25	0.43	0.44	0.43	0.42	0.4	0.39	0.39	0.38	0.38	0.38	0.37	0.37
26	0.35	0.37	0.36	0.34	0.33	0.32	0.31	0.31	0.31	0.3	0.3	0.3
27	-	0.31	0.3	0.27	0.27	0.26	0.26	0.25	0.25	0.25	0.24	0.24

References

31. Baskerville, G.L.; Emin, P. Rapid Estimation of Heat Accumulation from Maximum and Minimum Temperatures. *Ecology* **1969**, *50*, 514–517, doi:10.2307/1933912.
32. Poliak, S.S. [Changes in the indicators of immunologic reactivity in patients with juvenile uterine hemorrhages in different methods of therapy]. Akush Ginekol (Mosk) **1975**, 29–31.
33. Therneau, T. A Package for Survival Analysis in R. *R package* **2015**.
34. Yang, H.M.; Macoris, M.L.G.; Galvani, K.C.; Andrighetti, M.T.M.; Wanderley, D.M.V. Assessing the Effects of Temperature on the Population of Aedes Aegypti, the Vector of Dengue. *Epidemiology & Infection* **2009**, *137*, 1188–1202, doi:10.1017/S0950268809002040.
35. Padfield, D.; O'Sullivan, H.; Pawar, S. rTPC and Nls.Multstart: A New Pipeline to Fit Thermal Performance Curves in r. *Methods in Ecology and Evolution* **2021**, *12*, 1138–1143, doi:10.1111/2041-210X.13585.
36. H Sparks, A.; Hengl, T.; Nelson, A. GSODR: Global Summary Daily Weather Data in R. *JOSS* **2017**, *2*, 177, doi:10.21105/joss.00177.
